# The Aqueous Stem Bark Extract of *Alstonia boonei* Exhibits Anticataract Activity in Sprague Dawley Rat

**DOI:** 10.1155/2023/5524137

**Published:** 2023-08-01

**Authors:** Adwoa Frema Amanfo, Samuel Kyei, Yaw Duah Boakye, Clement Osei Akoto, Justice Kwaku Addo, Kofi Oduro Yeboah, Newman Osafo

**Affiliations:** ^1^Department of Pharmacology, Faculty of Pharmacy and Pharmaceutical Sciences, College of Health Sciences, Kwame Nkrumah University of Science and Technology, KNUST, Kumasi, Ghana; ^2^Department of Optometry and Vision Science, University of Cape Coast, Cape Coast, Ghana; ^3^Biomedical and Clinical Research Centre, University of Cape Coast, Cape Coast, Ghana; ^4^Department of Pharmaceutics, Faculty of Pharmacy and Pharmaceutical Sciences, College of Health Sciences, Kwame Nkrumah University of Science and Technology, KNUST, Kumasi, Ghana; ^5^Department of Chemistry, College of Science, Kwame Nkrumah University of Science and Technology, KNUST, Kumasi, Ghana; ^6^Department of Chemistry, University of Cape Coast, Cape Coast, Ghana

## Abstract

In Africa, *Alstonia boonei* is used folklorically for the management of the multitude of conditions including cataract, which accounts for 50% of cases of blindness in the region. The current study set out to probe the traditional use of the aqueous extract of *Alstonia boonei* stem bark (ABE) as an anticataract remedy using Sprague Dawley rat models. We investigated the probable phytochemical constituents in the extract, *in vitro* antioxidant potential, and its *in vitro* aldose reductase inhibition. For the anticataract investigations, diabetic cataract was induced using galactose in 3-week-old Sprague Dawley rats, and age-related cataract was induced by the administration of sodium selenite to 10-day-old rat pups. Cataract scores in both models were determined after treatment with 30, 100, and 300 mgkg^−1^ doses of ABE and 10 mlkg^−1^ of distilled water. Lens glutathione, total lens protein, soluble lens proteins (alpha-A) crystallin, and aquaporin 0 levels in the enucleated lens homogenates were determined. Changes in lens to body weight were also determined with histopathological analysis done on the lenses in the selenite-induced cataract model. The presence of alkaloids, tannins, flavonoids, glycosides, and triterpenoids was identified in the extract. The extract inhibited aldose reductase activity with IC_50_ of 92.30 *μ*gml^−1^. The 30, 100, and 300 mgkg^−1^ABE-treated rats recorded significantly (*p* < 0.05) reduced cataract scores indicating a delay in cataractogenesis in galactose-induced cataract and in selenite-induced cataractogenesis as well. Markers of lens transparency such as AQP0, alpha-A crystallin, and total lens proteins and lens glutathione levels were significantly (*p* < 0.05) preserved. In conclusion, this study establishes the anticataract potential of the aqueous stem bark extract of *Alstonia boonei* in Sprague Dawley rat models.

## 1. Introduction

Cataracts are described as lens clouding that results in visual impairment [1, 2]. In Africa, it accounts for about 50% of cases of blindness [3, 4] and 54.8% of the cases in Ghana [5]. Cataract can be congenital and age-related or may be a result of underlining diseases such as diabetes and is therefore characterised as a disease of priority.

Age-related cataract remains a leading cause of blindness worldwide and is believed to increase the risk of death [6]. Multiple mechanisms are involved in the development of diabetic cataract. Earlier studies have identified the lens' response to hyperglycaemic environment through processes such as increased oxidative stress [7] which plays a major part in the pathogenesis of diabetic cataract.

To eliminate cataract, the WHO launched the Vision 2020 campaign [1], which culminated in the realisation that individuals who patronised cataract surgery were much less than expected, i.e., 523 out of 2000 patients [8]. This means that significant proportions of people suffering from cataracts do not seek medical help or resort to alternative treatment options other than surgery [9, 10]. As such, the effectiveness and relevance of these alternative treatment options in the management of cataracts need to be investigated.

One of the known alternatives to surgery largely employed in cataract treatment in Africa is the use of plant and plant-based products [11]. One such plant extensively used in the management of cataract in Africa is *Alstonia boonei* although there has not been any scientific justification of its use in that regard.

*Alstonia boonei* De Wild (Apocynaceae) is a large tree commonly found in the dry lowlands and rainforests across West Africa. The stem bark has a myriad of uses in folklore medicine, including its use as antimalarial and antivenom [12] and anticataract [13]. The current study, therefore, set out to investigate the anticataract activity of aqueous extract of the stem bark of *Alstonia boonei* in galactose- and selenite-induced cataracts in Sprague Dawley rats and provide scientific evidence to its traditional use.

## 2. Materials and Methods

### 2.1. Plant Collection

*Alstonia boonei* stem bark was collected from Asakraka-Kwahu in the Eastern Region of Ghana in November 2020 and validated at the Department of Herbal Medicine, KNUST (Voucher specimen No: KNUST/HMI/2022/SB002). The plant material was then sun-dried.

### 2.2. Animals

Sprague Dawley rats (3 weeks old and 10 days old) of both sexes were procured from the Center for Plant Medicine Research (CPMR), Mampong, Eastern Region, Ghana, and housed in stainless steel cages at the Department of Pharmacology Animal Housing Facility, UCC. Rats were allowed free access to commercial chow and water. Animals were humanely handled throughout the entire duration of the studies.

### 2.3. Chemicals

Acetic anhydride, ammoniacal alcohol, ammonia (liquid), chloroform, Dragendorff's reagent, hydrochloric acid, Mayer's reagent, Fehling's solution A &B, ferric chloride, lead acetate, reduced nicotinamide adenine dinucleotide phosphate (NADPH), sodium selenite, sulphuric acid, and sodium hydroxide were purchased from Sigma-Aldrich, Germany; galactose was purchased from ACROS, New Jersey, USA; tropicamide ophthalmic solution (1%) was purchased from Alcon Laboratories South Africa (Pty) Ltd.

### 2.4. Extraction

About 640 g of the stem bark of *Alstonia boonei* was blended using a heavy-duty blender (37BL85 (240CB6) Waring Commercial, USA). The fine powder obtained was transferred into a percolator and macerated with 4.5 L of distilled water for 3 days. The mixture was filtered, and the filtrate was concentrated at 40°C using a rotary evaporator (Rotavapor R-210, BUCHI, Switzerland) and further concentrated at 50°C using an industrial oven (Gallenkamp OMT Oven, SANYO, Japan). A brownish solid is produced which was stored at 4°C, reconstituted using PBS when needed, and referred to as *Alstonia boonei* extract (ABE) [14].

### 2.5. Preliminary Phytochemical Screening

The phytochemical contents of ABE were studied using methods as described by Harborne [15].

### 2.6. *In Vitro* Antioxidant Assays

#### 2.6.1. Total Flavonoid Content

The method described by Chang et al. [16] was used. Briefly, a mixture of 0.5 ml of the extract in 0.3 ml of 5% NaNO_2_ and 0.3 ml of 10% AlCl_3_ was prepared. The mixture was incubated at 25°C for 30 min in a laboratory incubator (Yohmai IN-601, Stains, France), 2 ml of 1 moll^−1^ NaOH was added, and absorbance was determined at 415 nm using a spectrophotometer (BioTek-800-TS absorbance reader, Agilent, Santa Clara, USA). A standard curve was established with quercetin, and the total flavonoid capacity of the extract was extrapolated from the curve.

#### 2.6.2. Total Phenolic Content

The Folin–Ciocalteu method as described by Hudz et al. [17] was used in this determination.

A 0.6 ml mixture containing 0.1 ml of 0.5 N Folin–Ciocalteu and 0.5 ml of ABE was prepared and incubated at 25°C for an hour, and 2.5 ml of 2% NaHCO_3_ was added afterwards. The resulting solution was further incubated at ambient temperature for 90 min, and the absorbance was determined at 760 nm using a spectrophotometer (BioTek-800-TS absorbance reader, Agilent, Santa Clara, USA).

#### 2.6.3. Total Antioxidant Capacity

Total antioxidant capacity estimated as gallic acid equivalent (GAE) was determined as earlier described by Urbano et al. [18]. A final mixture containing 1 ml of ABE and 3 ml of a solution made up of sulphuric acid, disodium phosphate, and ammonium molybdate at concentrations 0.6 M, 28 mM, and 4 mM, respectively, was incubated (Yohmai IN-601, Stains, France) at 95°C for 90 min. The absorbance of the mixture was determined at 695 nm (BioTek-800-TS absorbance reader, Agilent, Santa Clara, USA) upon cooling. Gallic acid was used to establish a standard curve with the GAE of ABE being extrapolated from the obtained curve.

### 2.7. Free Radical Scavenging Activity

The method described by Sharma and Bhat [19] was used in this determination. Briefly, 1 ml of varying concentrations of ABE (i.e., 2000−62.5 *μ*gml^−1^ in methanol) was added to a 20 mgl^−1^ mixture of methanol and DPPH. The resulting solution was incubated (Yohmai IN-601, Stains, France) under shade for 30 min at room temperature, and the absorbance was determined afterwards at 517 nm (BioTek-800-TS absorbance reader, Agilent, Santa Clara, USA), using ascorbic acid (i.e., 100−0.78 *μ*gml^−1^) as the positive control. The IC_50_, the concentration at which there was a 50% decrease in the absorbance of DPPH, was then determined using the relation:

(1) $$\begin{array}{c} \%DPPH\;scavenging\;ability=\left(\frac{absorbance\;of\;control-absorbance\;of\;ABE}{absorbance\;of\;control}\right)x\;100\%\text{.} \end{array}$$

### 2.8. Aldose Reductase Inhibition Assay

In this assay, unrefined aldose reductase (AR) in enzyme solution was collected as the supernatant from a homogenized rat lens which was centrifuged (Bioswisstec Hermle *Z* 36HK, Schaffhausen, Switzerland) at 5000 × g for 30 min at 4°C [20]. Varying concentrations of quercetin (control) and ABE (10–1000 *μ*gml^−1^) were prepared as inhibitors of AR activity using phosphate-buffered saline (PBS). To 50 *μ*l of the enzyme solution in a cuvette, 50 *μ*l ABE, 50 *μ*l 0.1 M phosphate buffer (pH 7), and 50 *μ*l 0.03 mM NADPH were added. The enzyme reaction was initiated with the addition of 50 *μ*l D-xylose to the mixtures, and absorbance was determined at 340 nm after 180 s using BioTek 800 TS absorbance reader (Agilent, Santa Clara, USA). The inhibition of AR activity was expressed as a percentage of quercetin [21].

### 2.9. Galactose-Induced Cataractogenesis

The method used by Kyei et al. [22] and modified by Amanfo et al. [23] was employed in this study. Briefly, 3-week-old Sprague Dawley rats had their lenses examined for cataractogenesis using the slit lamp guided by the graded score and put into five groups (*n* = 5) and treated as indicated below for 6 weeks: Group 1 received only 10 mlkg^−1^ distilled water p.o. for 6 weeks; Group 2 received 3000 mgkg^−1^ galactose p.o. daily plus 10 mlkg^−1^ distilled water p.o. for 6 weeks; and Groups 3–5 received 3000 mgkg^−1^ galactose p.o. daily plus 30, 100, or 300 mgkg^−1^ ABE p.o. for 6 weeks, respectively.

The lenses of all rats used in the study were examined for degree of opaqueness weekly using a Macro II-B Slit Lamp (Marco-Lombart Instrument, Japan) and scored based on the scale detailed (Table 1) as described by Amanfo et al. [23].

### 2.10. Blood Sugar Determination

Blood samples were taken, before the start of treatment and weekly, from the rat tail vein. All samples were taken after an overnight fast, and fasting blood sugar levels were determined using a glucometer (Accu Chek Performa, Roche Diagnostics, USA).

### 2.11. Lens-to-Body Weight Ratio Determination

Rats were weighed before the study, and the lens was also weighed when extracted at the end of the study. The lens-to-body weight ratio was then computed.

### 2.12. Lens Glutathione (GSH) Assay

The extracted lenses were homogenized in PBS and centrifuged (Bioswisstec Hermle Z 36HK, Schaffhausen, Switzerland) at 5000 × g for 30 min to obtain a supernatant used for subsequent determinations. The supernatant obtained was used to determine GSH levels in the lens using a glutathione ELISA kit (Shanghai, China) in accordance with the guidelines provided by the manufacturer. The measurements were made in duplicate.

### 2.13. Total Lens Protein Assay

Using a bicinchoninic acid (BCA) ELISA kit purchased from Shanghai Chemical Ltd, China, we determined the lens total protein from the supernatant using the manufacturer's guidelines. The measurements were made in duplicate.

### 2.14. Selenite-Induced Cataractogenesis

We employed the model detailed by Kyei et al. [24] and modified by Amanfo et al. [23] in this investigation. The 10-day-old rats were given subcutaneous shots of 15 *μ*molkg^−1^ sodium selenite daily for two days and put into five groups (*n* = 4), and treatments meted out are detailed as follows: Group 1 received only 10 mlkg^−1^ distilled water p.o. and 12 hourly for 21 days without any sodium selenite being given to the rats; Group 2 received only 10 mlkg^−1^ distilled water p.o. 30 min before the sodium selenite challenge and 12 hourly for 21 days; and Groups 3–5 received 30, 100, and 300 mgkg^−1^ ABE p.o. 30 min before the sodium selenite challenge and 12 hourly for 21 days, respectively.

The pupils of the pups were dilated via ophthalmic tropicamide installation on day 22, and lenses were accessed for cataractogenesis using the slit lamp.

### 2.15. Cataract Grading

The extent of cataract was graded as described by Sippel [25] and is detailed in Table 2.

### 2.16. Lens Soluble Proteins (CRYAA) and Aquaporin 0 (AQP0) Levels

Rat lenses were extracted at the end of the study and homogenized in PBS. The resultant solution was centrifuged (Bioswisstec Hermle *Z* 36HK, Schaffhausen, Switzerland) at 5000 × g for 30 min, and the supernatant was collected for CRYAA and AQP0 assays using ELISA kits (Shanghai Chemical Ltd, China) in accordance with the manufacturer's guidelines. The measurements were all done in duplicate, and the experiment was repeated three times.

### 2.17. Histopathological Analysis

Extracted lenses were fixed in 10% phosphate-buffered paraformaldehyde and wax-infiltrated paraffin. Sections were prepared and stained with haematoxylin and eosin for histopathological analysis [26].

### 2.18. Statistical Analysis

Results were plotted and analyzed using GraphPad Prism for Windows 8.0.1 (GraphPad Software Inc., USA). Values were considered statistically significant at *p* < 0.05.

## 3. Results

### 3.1. Phytochemical Screening

ABE was realized to have detectable levels of alkaloids, flavonoids, glycosides, tannins, and triterpenoids (Table 3).

### 3.2. *In Vitro* Antioxidant Assay

**Figure 1:**
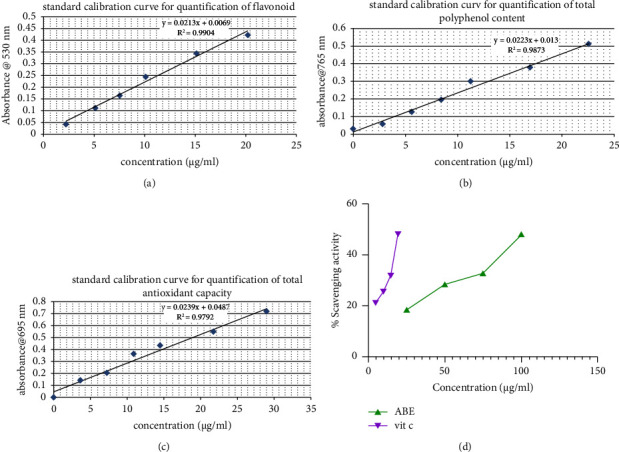
Standard calibration curve for flavonoid (a), total phenols (b), and antioxidant (c) quantification and free radical scavenging activity of ABE (d).

There was an observable increase in the total antioxidant capacity of all standards used with increased concentrations of ABE (Figures 1(a)–1(c)). The extract was realized to exhibit a dose-dependent scavenging activity (Figure 1(d)), and the standards of 725.43, 663.49, and 534.85 *μ*gg^−1^ were realized for vitamin C, gallic acid, and quercetin, respectively.

### 3.3. Aldose Reductase Inhibition Assay

In this assay, ABE was realized to show an inhibition of aldose reductase activity with an IC_50_ of 92.30 *μ*gml^−1^ (Table 4).

### 3.4. Galactose-Induced Cataractogenesis

#### 3.4.1. Effect of ABE on Blood Sugar Levels

**Figure 2:**
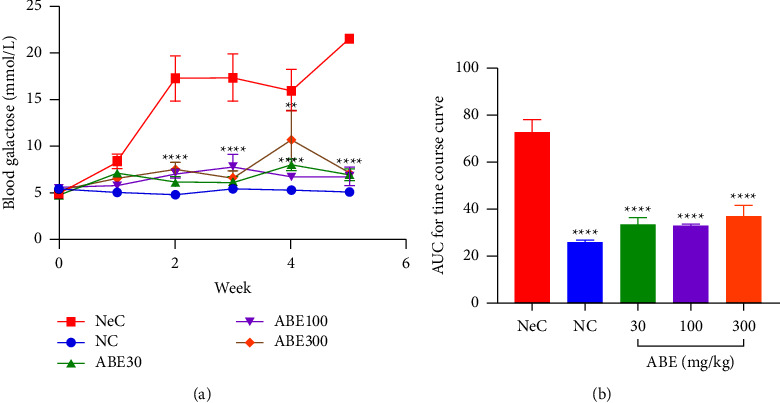
Effect of *Alstonia boonei* aqueous extract on blood galactose levels in Sprague Dawley rats: (a) the time course and (b) the area under the time-dependent curves (AUC). ^*∗∗*^*p* < 0.01, ^*∗∗∗∗*^*p* < 0.0001 significance between the negative control (NeC) group and the other treatment groups (2-way ANOVA followed by Tukey's post hoc test in (a); ANOVA followed by Dunnett's post hoc test in (b); means ± SEM (*n* = 5).

From the time-course curve, the normal control (NC) rats were observed to show a steady level of blood galactose over the 5-week period. Upon galactose administration to rats, there was a steady rise in blood galactose level in the rats in the negative control (NeC) group. Treatment with ABE resulted in a significant (*p* < 0.05) drop in galactose levels at all dose levels studied over the 5-week period (Figure 2(a)).

The area under the time-course curve (AUC) confirmed the observed blood galactose levels of 26.32 ± 0.55 in NC rats which increased to 72.59 ± 4.996 upon galactose administration in NeC rats. This rise in blood sugar was significantly reduced by ABE administration at doses of 30,100, and 300 mgkg^−1^ to levels of 33.85 ± 2.85, 33.62 ± 0.20, and 37.95 ± 3.30, respectively (Figure 2(b)).

#### 3.4.2. Effect of ABE on Galactose-Induced Cataractogenesis

**Figure 3:**
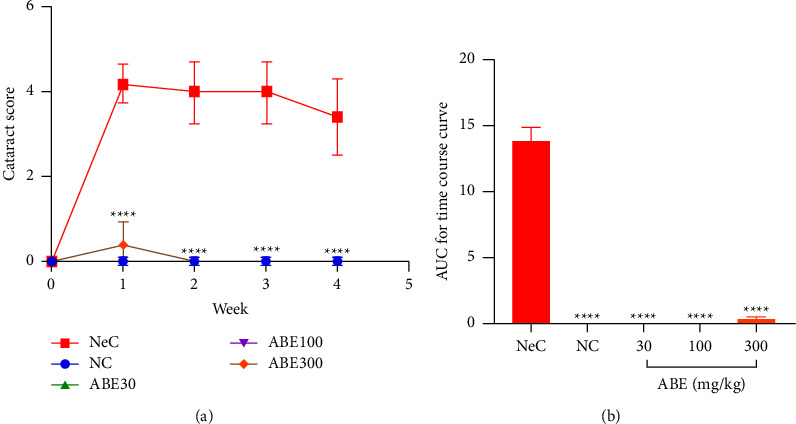
Effect of *Alstonia boonei* aqueous extract on cataractogenesis in Sprague Dawley rats: (a) the time-course and (b) the area under the time-dependent curves (AUC). ^*∗∗∗∗*^*p* < 0.0001; significance between the negative control (NeC) group and the other treatment groups (2-way ANOVA followed by Tukey's *post hoc* test in (a); ANOVA followed by Dunnett's *post hoc* test in (b); means ± SEM (*n* = 5).

From the time-course curves obtained, galactose administration to rats caused an increase in cataract score which peaked after 1 week. However, ABE administration caused practically undetectable signs of cataractogenesis comparable with determinations made in NC rats (Figure 3(a)).

When the AUCs were determined, it established cataract score values spanning from 0 to 2 for ABE-treated rats as compared to about 13 in NeC rats (Figure 3(b)).

#### 3.4.3. Effect of ABE on Lens-to-Body Weight Ratio

**Figure 4:**
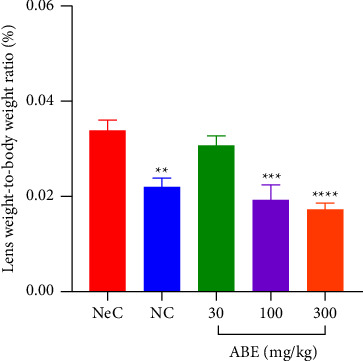
Effect of *Alstonia boonei* aqueous extract on the lens-to-body weight ratio of Sprague Dawley rats with galactose-induced cataract. ^*∗∗*^*p* < 0.01; ^*∗∗∗*^*p* < 0.001; ^*∗∗∗∗*^*p* < 0.0001, significance between the negative control (NeC) group and the other treatment groups (ANOVA followed by Dunnett's post hoc test). The values plotted are the mean ± SEM (*n* = 5).

The lens-to-body weight ratio was realized to significant (*p* < 0.05) increase in control rats exposed to galactose as compared to the distilled water-treated rats. Upon ABE treatment, rats recorded a significant (*p* ≤ 0.001) reduction in the lens-to-body weight ratio at 100 and 300 mgkg^−1^ doses administered (Figure 4).

#### 3.4.4. Effect of ABE on Lens Glutathione (GSH) and Protein Levels

**Figure 5:**
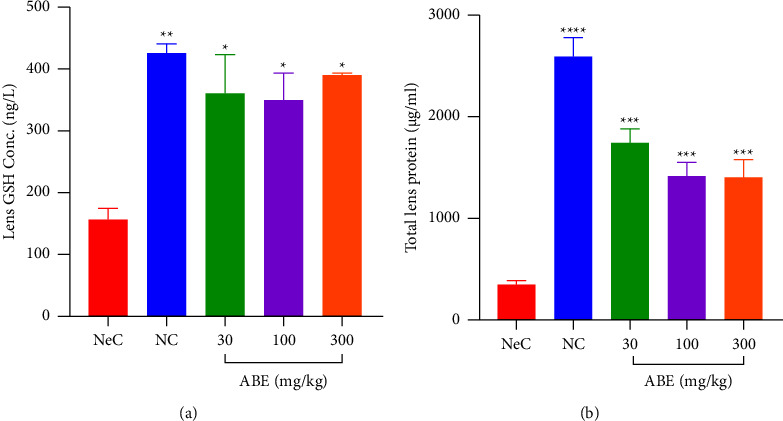
Effect of *Alstonia boonei* aqueous extract on lens GSH and total protein levels of Sprague Dawley rats with galactose-induced cataract. ^*∗∗*^*p* < 0.05; ^*∗∗*^*p* < 0.01, ^*∗∗∗*^*p* < 0.001; ^*∗∗∗∗*^*p* < 0.0001, significance between the negative control (NeC) group and the other treatment groups (ANOVA followed by Dunnett's post hoc test). The values plotted are the mean ± SEM (*n* = 5).

In this study, the lens glutathione levels were determined to be in 427.8 ± 12.49 ngl^−1^ NC rats which reduced significantly (*p* < 0.05) to 160.4 ± 13.71 ngl^−1^ when rats were challenged with galactose. Upon ABE administration, the GSH levels were significantly (*p* < 0.05) increased to 363.2 ± 58.73, 349.9 ± 42.61 ngl^−1^, and 392.1 ± 1.933 ngl^−1^ at doses of 30, 100, and 300 mgkg^−1^, respectively (Figure 5(a)).

Similarly, the total lens proteins of 2609 ± 186.1 *μ*gml^−1^ realized in NC rats were significantly lowered after the galactose challenge to 347.2 ± 44.10 *μ*gml^−1^. However, when ABE was administered to rats at 30, 100, and 300 mgkg^−1^, there were significant increases in the total lens protein levels to 1756 ± 131.1, 1415 ± 144.2, and 1400 ± 185.6 *μ*gml^−1^ (Figure 5(b)).

### 3.5. Selenite-Induced Cataractogenesis

#### 3.5.1. Effect of ABE on Selenite-Induced Cataractogenesis

**Figure 6:**
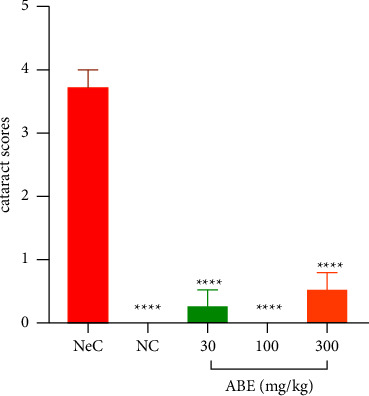
Effect of *Alstonia boonei* aqueous extract on the cataract score in Sprague Dawley rat pups with selenite-produced cataract. Comparison between the NeC and treatment groups: ^*∗∗∗∗*^*p* < 0.0001 (ANOVA followed by Dunnett's *post hoc* test). The values plotted are the mean ± SEM (*n* = 5).

Upon slit lamp analysis, it became apparent that NC rats developed no cataract over the duration of the study. 6 out of 8 eyes of rats in NeC showed grade IV cataract with the other 2 showing grade III nuclear cataract. Upon treatment with ABE, grade I cataract was only observed in 2 eyes in 30 mgkg^−1^ treated rats, 0 cataract recorded in rats treated with 100 mgkg^−1^ of extract, and 3 eyes in 300 mgkg^−1^ treated rats showed grade I cataract (Figure 6).

#### 3.5.2. Effect of ABE on Lens Soluble Proteins (CRYAA) and Aquaporin 0 (AQP0)

**Figure 7:**
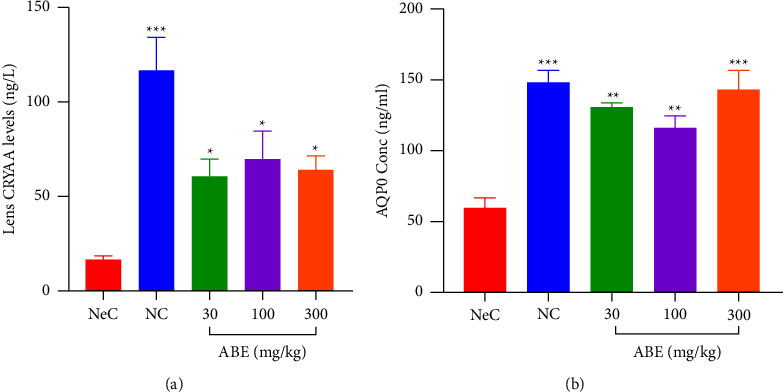
Effect of *Alstonia boonei* aqueous extract on lens soluble protein concentration (a) and aquaporin 0 (b) levels in Sprague Dawley rat pups with selenite-induced cataract. Comparing NeC and treated groups: ^*∗*^*p* < 0.05, ^*∗∗*^*p* < 0.01, ^*∗∗∗*^*p* < 0.001 (ANOVA followed by Dunnett's post hoc test). The values plotted are the mean ± SEM (*n* = 5).

In the CRYAA assay, NC rats recorded 116.4 ± 17.69 ngl^−1^ which was significantly (*p* < 0.05) decreased when rats were challenged with selenite to produce 15.43 ± 3.005 ngl^−1^ in NeC. When ABE (30, 100, and 300 mgkg^−1^) was administered, CRYAA levels were significantly (*p* < 0.05) increased to 59.76 ± 9.720 ngl^−1^, 69.66 ± 14.55 ngl^−1^, and 63.94 ± 7.361 ngl^−1^, respectively (Figure 7(a)).

In a similar manner, NC rats recorded AQP0 levels of 61.34 ± 5.559 ngml^−1^ which was significantly (*p* < 0.05) decreased when rats were challenged with selenite. When ABE (30, 100, and 300 mgkg^−1^) was administered, AQP0 levels were significantly (*p* < 0.05) increased to 132.4 ± 2.163 ngml^−1^, 117.3 ± 8.140 ngml^−1^, and 144.7 ± 12.62 ngml^−1^, respectively (Figure 7(b)).

### 3.6. Histopathological Analysis

**Figure 8:**
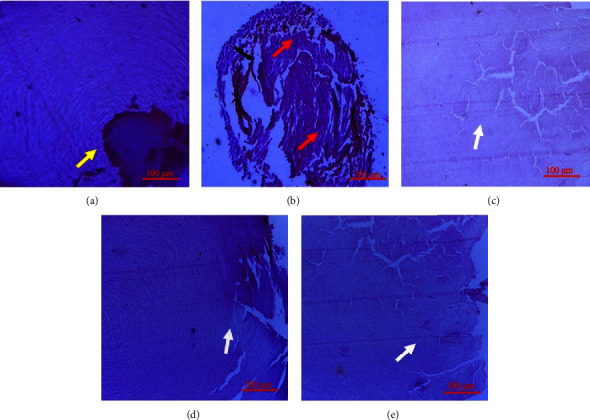
Photomicrographs of lenses: (a) normal lenses (normal control); (b) negative control lenses; (c) selenite-induced lens cataract treated with 30 mgkg^−1^ ABE; (d) selenite-induced lens cataract treated with 100 mgkg^−1^ of ABE; (e) selenite-induced lens cataract treated with 300 mgkg^−1^ ABE. Yellow arrow: regular arrangement of lens fibres in the lens cortex; red arrow: lens epithelial erosion and abnormal morphology of lens fibres; white arrow: preserved lens integrity and normal architecture of lens fibres. The micron bar represents 100 *μ*m.

The histopathology of NC rats presented a regular arrangement of lens fibres in the cortex (yellow arrow) (Figure 8(a)). In NeC rats with full-blown cataract, there were observable signs of lens epithelial erosion and abnormal changes in lens fibre morphology (red arrows). Again, NeC rats exhibited clear signs of distorted lens fibres impregnated with fragmentations (Figure 8(b)). In rats treated with ABE, there were no clear signs of disturbance to the lens fibre integrity and architecture (white arrow) (Figures 8(c)–8(e)).

## 4. Discussion

Current understanding of the precise mechanism involved in the progression of diabetic cataract is still limited, leaving significant gaps in our knowledge. As a result, the development of reliable preventive and therapeutic medications for this condition is yet to be achieved. Nevertheless, the galactose cataract model replicates the occurrence of secondary cataract. Compared to glucose-induced cataract, galactose-induced cataract is efficient. The model is economical and can rapidly replicate the pathophysiology of diabetic cataract. Oftentimes, galactosemic cataract is used to investigate the mechanisms of action of drugs employed in the management of diabetes-associated complications. Moreover, galactosemic cataract develops quickly and is reversible compared to other models of diabetic cataract, such as streptozotocin and alloxan [27].

Galactosemic cataract mimics diabetic cataracts and is characterised by the detection of trace amounts of sugar in peripheral blood following the administration of galactose. These traces of sugar are instrumental to cataract development. On the other hand, selenite shots in the selenite cataract model, a replicate of age-related cataract, cause cataractogenesis by increasing oxidative stress due to increased levels of reactive oxygen species [28] and reduced solubility of soluble proteins such as *α*, *β*-*γ* crystallin [29] and distortion of calcium balance [30].

In the galactose cataract model, increased galactose consumption results in hyperglycemia which in a cascade reaction leads to upregulation of aldose reductase [31, 32], the enzyme involved in the polyol pathway. Aldose reductase causes galactose to be metabolized into galactitol [33]. Galactitol accumulates in the lens because it cannot cross the lens membranes by passive diffusion, leading to the increased osmotic pressure of the lens and lens swelling and an increase in weight [34]. In addition, there is an increase in the expression of reactive oxygen species that can also destroy the lens through oxidative stress [35].

In contrast to the low affinity for glucose, aldose reductase has a higher affinity for galactose. Additionally, galactitol, the alcohol metabolite of galactose, has been shown to be much more challenging for sorbitol dehydrogenase to metabolize compared to sorbitol. Thus, galactosemia is more likely to cause severe cataracts in shorter time periods [36, 37]. With all these disparities, the extract was able to significantly decrease sugar levels, effectively preventing hyperglycaemia and development of cataracts.

Moreover, *Alstonia boonei* extract decreased cataract scores in rats. This observed effect can be attributed to the extract's inhibitory effect on aldose reductase, as well as the ability of the extract to prevent potential oxidative stress caused by the build-up of ROS when galactitol accumulates in the lens. In addition, this assertion is supported by the absence of physical signs of cataract, including an increase in lens weight and loss of body weight [23]. However, a limitation of this finding was our inability to estimate galactitol levels in addition to the measurement of blood glucose levels.

Among the various antioxidants present in the lens, the tripeptide glutathione is the most abundant and plays a crucial role in the preservation of lens transparency [38]. Furthermore, a decrease in total lens proteins is a recognized sign of precataractous changes [22]. Given this, the extract's ability to increase both glutathione and lens protein levels contributed to the improvement in lens transparency observed in the extract-treated rats.

In addition to the *Alstonia boonei* extract's inhibition of galactose-induced cataract, treatment with the extract also suppressed selenite-induced cataract in rat pups. This model serves as a valuable model for studying senile cataracts. It offers several advantages, including the ability to rapidly and consistently induce cataract formation within a short timeframe [39]. Due to similarities observed in increased levels of calcium and insoluble proteins, reduced levels of glutathione, and vesicular formation, this model is well suited for investigating the fundamental mechanisms that underpin human cataract formation [40]. Signs of sodium selenite-induced cataracts include m-calpain activation, which results in increased calcium levels through a cascade of biochemical processes. Additionally, there is an elevation in insoluble proteins, oxidative stress, and a reduction in lens soluble proteins such as *α*, *β*-*γ* crystallin [41].

The preventive and therapeutic effects of phytoconstituents in age-related ocular disorders, particularly cataracts, have been extensively documented [42]. Flavonoids have demonstrated protective effects against opacification of the lens through inhibition of glycoxidation [43, 44]. Likewise, alkaloids have been found to counteract oxidative damage induced by ROS like hydrogen peroxide [45]. The presence of these phytoconstituents in the extract may contribute to its ability to prevent cataract formation.

Additionally, the extract demonstrated a preventive or delaying effect on selenite-induced cataracts in rodent pups. This outcome holds clinical significance as delaying the onset of cataract formation can help prevent the associated visual impairment that can impact the independence of affected individuals [46, 47]. In fact, even a relatively short delay in cataract development can significantly improve the quality of life, reduce reliance on others, and prolong survival by about 10 years [48]. The observed delay seen in the low- and high-dose treatment has the potential to significantly improve the lives of individuals, particularly the elderly, over a considerable period of time.

The impact of oxidative stress on selenite-induced cataracts is evident from the rapid emergence of nuclear cataracts within a maximum of 5 days following sodium selenite administration. Secondary metabolites from plants such as flavonoids and tannins possess antioxidant properties that enable them to eliminate ROS [49]. The presence of these phytochemicals in *Alstonia boonei* extract could explain its preventive effect on senile cataract development.

Senile cataracts involve a cascade of biochemical processes, including a general reduction in protein levels and the insolubilization of soluble proteins within the eye lens [50]. Proteins serve as essential transport channels, and in the case of selenite-induced cataracts, the transport system within the lens is compromised [51, 52]. Proteins such as alpha-A crystallin function as a molecular chaperone in the lens [53–55], whereas others such as aquaporin 0 (AQP0), also known as main intrinsic polypeptide (MIP), serve as a water channel [56]. Treatment with the extract maintained the levels of these markers within the lens, which likely contributed to the observed anticataract effect.

## 5. Conclusions

The aqueous stem bark extract of *Alstonia boonei* delays and prevents galactose and selenite-induced cataractogenesis in Sprague Dawley rats and pups, respectively. The observed anticataract activity may be attributed to the extract's ability to decrease oxidative stress in the lens. The current study gives scientific evidence to its claim as an anticataract agent. Future studies may, however, identify the specific phytochemical(s) accounting for the probable anticataract effect of the aqueous stem bark extract of *Alstonia boonei*.

## Figures and Tables

**Table 1:** Assessment of degree of lens opaqueness.

Score	Description
0	Clear lens with no presence of vacuoles
1	Clear lens with less than 3 vacuoles
2	Clear lens with more than 3 vacuoles
3	Vacuoles covering entire lens surface
4	Incomplete opacity of lens
5	Complete lens opacity

**Table 2:** Cataract classification.

Score	Description
0	Clear lens
1	Distended lens fibres with subcapsular opacity
2	Distended lens fibres in the cortex with observable nuclear cataract
3	Peri-nuclear area opacity of lens with intense nuclear cataract
4	Total lens opacity

**Table 3:** Phytochemical screening of ABE.

	Tannins	Alkaloids	Flavonoids	Triterpenoids	Sterols	Saponins	Glycosides
ABE	+	+	+	+	—	—	+

**Table 4:** *In vitro* lens aldose reductase inhibitory effect of ABE.

Treatment	Concentration (*μ*gml^−1^)	% inhibition	IC_50_ (*μ*gml^−1^)
ABE	50	6.38	92.30
	100	21.21	
	200	26.56	
	400	26.97	
	800	33.86	

Quercetin	50	32.17	30.65
	100	41.14	
	200	44.57	
	400	45.8	
	800	47	

## Data Availability

The datasets generated and/or analyzed during the current study are available from the corresponding authors upon reasonable request.
